# The Major Brain Endocannabinoid 2-AG Controls Neuropathic Pain and Mechanical Hyperalgesia in Patients with Neuromyelitis Optica

**DOI:** 10.1371/journal.pone.0071500

**Published:** 2013-08-09

**Authors:** Hannah L. Pellkofer, Joachim Havla, Daniela Hauer, Gustav Schelling, Shahnaz C. Azad, Tania Kuempfel, Walter Magerl, Volker Huge

**Affiliations:** 1 Institute for Clinical Neuroimmunology, Ludwig-Maximilians University, Munich, Germany; 2 Department of Neurology, Ludwig-Maximilians University, Munich, Germany; 3 Department of Psychiatry and Psychotherapy, Georg August University, Göttingen, Germany; 4 Department of Anaesthesiology, Ludwig Maximilians University, Munich, Germany; 5 Chair of Neurophysiology, Center for Biomedicine and Medical Technology Mannheim (CBTM), Ruprecht Karls University Heidelberg, Mannheim, Germany; Southern Illinois University School of Medicine, United States of America

## Abstract

Recurrent myelitis is one of the predominant characteristics in patients with neuromyelitis optica (NMO). While paresis, visual loss, sensory deficits, and bladder dysfunction are well known symptoms in NMO patients, pain has been recognized only recently as another key symptom of the disease. Although spinal cord inflammation is a defining aspect of neuromyelitis, there is an almost complete lack of data on altered somatosensory function, including pain. Therefore, eleven consecutive patients with NMO were investigated regarding the presence and clinical characteristics of pain. All patients were examined clinically as well as by Quantitative Sensory Testing (QST) following the protocol of the German Research Network on Neuropathic Pain (DFNS). Additionally, plasma endocannabinoid levels and signs of chronic stress and depression were determined. Almost all patients (10/11) suffered from NMO-associated neuropathic pain for the last three months, and 8 out of 11 patients indicated relevant pain at the time of examination. Symptoms of neuropathic pain were reported in the vast majority of patients with NMO. Psychological testing revealed signs of marked depression. Compared to age and gender-matched healthy controls, QST revealed pronounced mechanical and thermal sensory loss, strongly correlated to ongoing pain suggesting the presence of deafferentation-induced neuropathic pain. Thermal hyperalgesia correlated to MRI-verified signs of spinal cord lesion. Heat hyperalgesia was highly correlated to the time since last relapse of NMO. Patients with NMO exhibited significant mechanical and thermal dysesthesia, namely dynamic mechanical allodynia and paradoxical heat sensation. Moreover, they presented frequently with either abnormal mechanical hypoalgesia or hyperalgesia, which depended significantly on plasma levels of the endogenous cannabinoid 2-arachidonoylglycerole (2-AG). These data emphasize the high prevalence of neuropathic pain and hyperalgesia in patients with NMO. The degree of mechanical hyperalgesia reflecting central sensitization of nociceptive pathways seems to be controlled by the major brain endocannabinoid 2-AG.

## Introduction

Neuromyelitis optica (NMO; Devic’s disease) is a demyelinating inflammatory autoimmune disease of the central nervous system leading to recurrent optic neuritis (ON) and episodic myelitis [1]–[3]. Comparable with multiple sclerosis (MS) it follows a relapsing course, but differs from MS in regard to clinical, radiologic, laboratory, and pathologic features. NMO is characterised by a longitudinally extensive spinal cord lesion spanning three or more vertebral segments, the lack of symptomatic brain lesions [3]–[5] and the presence of antibodies targeting the aquaporin-4 water channel (AQP4) in a significant proportion of patients, suggesting a B cell-mediated mechanism of disease [6]–[9]. In contrast to MS, disease attacks in patients with NMO are usually more severe and recover only partially.

Apart from the deterioration of visual and motor function, the presence of severe pain in a substantial proportion of NMO patients with a high impact on their health-related quality of life was only recently reported [10], [11]. However, the scale reflecting impairment of patients with multiple sclerosis (Expanded Disability Systems Score EDSS), which is also frequently used to characterize dysfunction in patients with NMO, does not include any measure reflecting pain or hyperalgesia [12]. Therefore, the presence of pain and the attending reduction in quality of life have been frequently overlooked and are underrepresented in most studies.

Although spinal cord inflammation is a defining aspect of NMO, there is still no knowledge of the pattern of somatosensory changes accompanying the disease, nor the degree of on going stress and depression frequently observed in NMO patients suffering from chronic pain. Thus, it is essential to characterise the complete somatosensory phenotype of patients with NMO, since this may contribute to a better understanding of the underlying pathophysiological mechanisms of pain generation in NMO, which may enable potential treatment options [13]–[15]. Quantitative sensory testing (QST) is a tool to characterise abnormalities of somatosensory function by controlled graded stimulation of all relevant subtypes of somatosensory afferents. The concise QST protocol employed in this study developed by the German Research Network on neuropathic pain (DFNS) [16]–[18], exhibits a high test-retest as well as high interobserver reliability [19] and is regarded as the currently most advanced QST method available [20]. Finally, as the endocannabinoid system is involved in peripheral and central pain control [21]–[23] and cannabinoid drugs acting at the CB1 receptor are approved for pain treatment in MS [24], we assessed the plasma levels of the two paradigmatic endocannabinoids acting at the CB1 cannabinoid receptor, 2-arachidonoylglycerol (2-AG) and arachidonoylethanolamide (anandamide, AEA) in all patients to identify a potential role of cannabinoids in endogenous pain control [25]–[27]. 2-AG was of particular interest, since it has the highest concentration of any brain lipid and is a major controller of synaptic transmission at physiological concentrations via inhibitory retrograde synaptic signalling and boosting of GABA-A receptor efficacy [28]–[30].

## Methods

### Patients

Eleven consecutive Caucasian patients with NMO seen at the Institute for Clinical Neuroimmunology were included into the study. All of them fulfilled diagnostic criteria of definite AQP4-antibody positive NMO [5]. The study was approved by the local ethics committee (ethics committee, medical faculty of the university of Munich, Germany), and written informed consent was obtained by all subjects enrolled in the study according to the Declaration of Helsinki. All necessary permits were obtained for the described study, which complied with all relevant regulations.

After given written informed consent nine female and two male patients with a median age of 51 years ranging from 24 to 69 were included (48.9±14.9 years; 9 female, 2 male). Median time since onset of disease was 8 years ranging from 3 months to 24 years (Table 1). Medical history was obtained in all patients; additionally previous MRI scans of the brain and the spinal cord were reviewed carefully in each patient. All patients were assessed clinically by EDSS score [12]. In addition, an age and gender-matched group of eleven healthy control subjects was assessed (48.8±13.8 years; 9 female, 2 male).

**Table 1:** Patient characteristics.

Pat	sex	disease duration (years)	age	last relapse (months)	EDSS	syringomyeliaactual ever		previous sMRI	actual sMRI	last cMR	immunotherapy
**1**	f	8	24	7	1,5	no	yes	Th3-9	small lesion Th3	normal	rituximab
**2**	f	22	61	26	8	yes	yes		atrophy whole spinal cord	normal	rituximab
**3**	f	24	69	10	6,5	yes	yes		Th1-8	nonspecific white matter	rituximab
**4**	f	8	27	18	2,5	no	no	C2-6;Th1-8	C4-6	lesion medulla oblongata	rituximab
**5**	f	3	48	8	2	no	yes	C6-Th6	Th2-4	nonspecific white matter	rituximab
**6**	f	5	46	3	1,5	no	no		Th1-3	bithalamic, medulla oblongata	None
**7**	f	7	51	9	7	yes	yes		small lesions whole spinal cord, atrophy below C7	ms typic white matter lesions, pons, medulla oblongata	rituximab
**8**	f	8	53	6	8	discrete	yes	C2-7; Th2-7, Th9	small lesions whole spinal cord, atrophy below Th7	nonspecific white matter, pons	rituximab +MTX
**9**	m	20	53	24	7	no	no		Th7-9, atrophy	normal	rituximab
**10**	m	4	67	27	3,5	no	no		Th3-8	optic neuritis	MMF
11	f	0,3	39	2,5	2	no	no	C2-7; Th3,5-6,8,10	Small lesions C3, C4, C6	Small lesion cerebellum	azathioprine

### Pain Assessment

The patients were interviewed for the presence, intensity, location, and characteristics of pain. Neuropathic pain was considered to be present if pain was (a) located in the area corresponding to CNS lesion, (b) could not be attributed to any other condition and (c) had specific characteristics of neuropathic pain according to the DN4 neuropathic pain scale [31], [32]. An 11 point numeric rating scale (NRS) scale ranging from 0–10 (0 = no pain, 10 = maximum conceivable pain) was used to assess the patientś subjective intensity of actual pain and maximum intensity of pain within the last three months.

### Biomarkers

Venous blood samples for endocannabinoid measurements were taken peripherally and drawn into Lithium-Heparin containing tubes (S-Monovette®, Sarstedt, Numbrecht, Germany), immediately centrifuged and then stored at –80°C to minimize ex-vivo synthesis of endocannabinoids from nucleated blood cells as has been described earlier [33]. Plasma concentrations of the endocannabinoids arachidonoylethanolamide (anandamide AEA) and 2-arachidonoylglycerol (2-AG) were determined within a few weeks after blood sampling using a method based on high performance liquid chromatography-tandem mass spectrometry (HPLC/MS-MS) as described elsewhere [34]. This method is linear within a range of 0.1 to 2 ng/ml for anandamide and 0.5 to 10 ng/ml for 2-AG. The inter-assay coefficient of variation is 34% for a mean anandamide concentration of 0.2 ng/ml and the lower limit of detection of the method (defined as a signal/noise ratio >4∶1) is 0.025 ng/ml for anandamide and 0.33 ng/ml for 2-AG. Because in biological matrices, 2-AG (including it’s deuterated analog) rapidly isomerizes to 1-AG [35] we quantified 2-AG as the sum of both isomers.

### Quantitative Sensory Testing

QST was conducted under minimal distraction in a silent, air-conditioned room, with an ambient temperature of 24–25°Celsius. QST followed the standardised protocol as described by the German Network on Neuropathic Pain (DFNS) [16]. All sensory tests were done bilateral on the dorsum of both hands as well as on the back of the feet. Total duration of sensory testing was about 120 minutes. In two patients QST testing had to be discontinued after testing of the feet, due to increasing pain and discomfort.

Thermal testing included Warm Detection Threshold (WDT), Cold Detection Threshold (CDT), Thermal Sensory Limen (TSL), Cold Pain Threshold (CPT), Thermal Sensory Limen (TSL), Cold Pain Threshold (CPT) and Heat Pain Threshold (HPT). Furthermore, Paradoxical Heat Sensations (PHS), that means identification of a cold stimulus as either hot or burning pain, were recorded. For mechanical testing we determined Mechanical Detection Threshold (MDT), Vibration Detection Threshold (VDT), Mechanical Pain Threshold (MPT), Mechanical Pain Sensitivity for pinprick stimuli (MPS), Wind-up Ratio (WUR), Pressure Pain Threshold (PPT), and Dynamic Mechanical Allodynia (DMA). For an elaborate discussion see [17].

### Psychological Assessment

The presence and severity of depression was assessed by the German version of the Center for Epidemiological Studies Depression Test (CES-D). [36], [37]. A raw test score of 27 or more is considered to be the critical limit for the presence of a depressive episode in pain patients [38].

Presence of stress symptoms such as sleep disturbances, nightmares and generalized irritability were measured using the German Version of the Post-Traumatic Stress Symptom 10-Questionnaire (PTSS-10) [39]. A summary score >35 is associated with a high probability of posttraumatic stress disorder (PTSD). Moreover, all patients completed a validated questionnaire evaluating different categories of traumatic memories including pain, nightmares, fear, and dyspnea occurring in the last week before the assessment [39].

Overall health-related quality of life (HRQL) was assessed by the German version of the Short Form 36 (SF-36). [40].

### Data Analysis

All QST data (except CPT, HPT, VDT and PHS) were transformed into decadic logarithms to achieve secondary normal distributions [16]. Paired t-test was used for group comparisons for data on interval level and to compare QST results of NMO patients with age and gender matched healthy control group. Additionally, data were further transformed into standard normal distributions (z-normalized) relative to reference data of the DFNS cohort of healthy subjects as well as for the healthy control group to allow comparison of sensory data across different QST parameters [17]. As described elsewhere, all patient data were normalized to the respective gender and age group of healthy controls using the equation: z = (individual value – mean _reference data base_)/SD_ reference data base_). The sign of the respective z-score was adjusted in a way that a gain of function was indicated by a positive sign, whereas a negative sign indicated a loss of function [15]. Significance of differences from healthy controls was estimated comparing the patients mean ± SD obtained by z-normalization vs. a standard normal distribution (i.e. mean ± SD = 0±1) of an equal number of healthy control subjects of the DFNS reference data and the age and gender-matched healthy controls using the web-based statistical freeware (Simple Interactive Statistical Analysis SISA, Uitenbroek 1997; http://home.clara.net/sisa/binomial.htm) [15]. Since QST exhibits a high test-retest and inter-observer reliability, it enables to compare QST results with the age and gender matched healthy controls of the DFNS [19]. The electronic analysis tool EQUISTA developed by the DFNS was used in order to subsume the frequency of abnormal sensory loss or sensory gain [15].

Data of SF36 were normalized as described above to a representative sample of the US-General population (n = 2393). In addition, correlations between QST results, endocannabinoid levels and the time since last relapse of NMO were calculated.

## Results

### Clinical Characteristics and MRI Findings

Clinical and MRI characteristics of NMO patients are shown in Table 1. Mean EDSS at the time of examination was 4.5 ranging from 1.5 to 8. Latency between examination and last relapse ranged from 2.5 to 27 months (mean 12.8, median 9 months). Spinal cord MRI showed extensive spinal cord lesion in all of them, however, in some patients lesions recovered partially or even completely during follow-up detected by repeated MRI. Syringomyelia was present in three patients and only discrete in one additional patient. Supraspinal MRI revealed normal brain MRI scan in 4 out of 11 patients, 4 patients had non-specific white matter lesions. Four patients showed lesions in the medulla oblongata or pons, one of them with additional bilateral thalamic lesions. Another patient had a small single lesion within the cerebellum.

### Pain Characteristics

Eight out of the eleven consecutive NMO patients (72%) reported on going pain (7 definite and 1 probable neuropathic pain according to [41]) at the time of examination. In 6 out of 11 patients, pain was even one of the predominant events during the last 7 days. Mean pain intensity at the time of examination was 4.2 (median 6) on an 11-point Likert scale ranging from 0 to 7. However, central neuropathic pain due to NMO was present in all except one patient (91% 9 definite, 1 probable) within the last three months, mean intensity was 7.3 ranging from 3 to 10 NRS (median 8). The only patient who did not suffer from central pain within the last three months had an episode of severe definite neuropathic pain during myelitis two years earlier, which was temporarily treated with gabapentin and recovered completely during the following months.

Most of the patients complained several different pain characteristics. The most frequently reported qualities of pain were burning, aching and pricking. Pain was located in the legs (n = 7), in the arms or shoulder (n = 5), in thoracic or abdominal dermatomes (n = 8) or combined (n = 7). Five patients had spontaneous on going pain as well as painful paroxysms; two patients complained of either solely attacks or constant pain, respectively (Table 2).

**Table 2:** Clinical characteristics of neuropathic pain.

Pat	pain actual (NRS)	max pain previous 6 months [NRS]	actual analgetic therapy	character of pain	location of pain	persisting pain	pain attacks
**1**	0	8	ibuprofen o. d.	pressure, lancinating, burning	upper back pain, shoulder, thoracal	no	yes
**2**	5	7	citalopram 10 mg, carbamazepin 300 mg,baclofen 25 mg, gabapentin 900 mg,	pressure, lancinating, burning	arms, abdominal region	yes	yes
**3**	6	8	mirtazapine 15 mg, tramadol 200 mg,gabapentin 1800 mg, carbamazepine1800 mg, diclophenac	burning,	legs	yes	no
**4**	6	10	ibuprofen o.d., amitriptyline 25 mg	lancinating	leg, lower back	yes (mild)	yes
**5**	0	0	none	burning, tingling	thoracical	no	no
**6**	0	6	none	lancinating	leg	no	yes
**7**	3	3	none	tingling, pressure	thoracical, belt	yes	no
**8**	6	9	pregabalin 300 mg, duloxetine 30 mg,morphine 2 mg	burning, tingling	legs, arms, thoracical	yes	no
**9**	7	10	carbamazepine 600 mg, ibuprofen 800 mg,tilidin 400 mg	burning, tingling, pruritic	legs	yes	yes
**10**	6	10	amitriptyline 25 mg, ibuprofen 400 mg	lancinating, tingling	back, thoracical, upper legs, abdominal, arms	yes	yes
**11**	7	10	amitriptyline 75 mg, pregabalin 300 mg,carbamazepine 400 mg	burning, tingling, lancinating	arms, legs, thoracical	yes	yes

Pain was treated continuously with analgesic or co-analgesic drugs in 7/11 patients (63%), all of whom needed more than one analgesic substance. One patient only took medication on demand during pain attacks and two more patients received antiepileptic drugs during previous relapses that where associated with pain but had recovered completely. Three patients administered a combination of sodium channel blocker to treat electric shooting pain attacks and a calcium channel blocker to reduce constant burning pain (Table 2).

### Stress, Depression and Health Related Quality of Life

Mean number of traumatic memories was 0.91; PTSS-10 score was 26.1±7.9. Three patients indicated no traumatic memories (PTSS-10∶18.3±4.2), 6 patients had one traumatic memory (27.8±7.9), while 2 patients displayed two traumatic memories (32.5±0.7). However, no patient displayed a PTSSD score>35, which would be firmly indicating comorbid posttraumatic stress disorder. Mean CES-D score was 34±8 indicating the presence of clinical relevant depression. Patients with NMO showed a significant decrease in three of the eight dimensions of SF 36, namely physical functioning, general health and noteworthy bodily pain (Table 3).

**Table 3:** Health related Quality of Life: (SF-36).

SF-36 Subscales	Mean±SD	Z-Score	p
PF	43.3±35.7	−1.8±1.6	<0.05
RP	52.5±43.2	−0.8±1.3	n.s.
BP	48.1±26.1	−1.1±1.1	<0.05
GH	42.6±14.7	−1.4±0.7	<0.05
VT	49.5±17.8	−0.6±0.8	n.s.
SF	79.5±17.9	−0.2±0.8	n.s.
RE	66.6±47.1	−0.4±1.4	n.s.
MH	65.4±14.5	−0.5±0.8	n.s.
PCS	34.1±10.2	–	
MCS	50.1±9.9	–	

The scales of the SF-36 score from 0–100, with 0 indicating worst health and 100 the best.

PF: Physical Functioning, RP: Role Limitations, Physical:, BP: Bodily Pain, GH: General Health, VT: Vitality, SF: Social Functioning, RE: Role Limitations, Emotional, MH: Emotional Well-Being, PCS: Physical Component Summary Score MCS: Mental Component Summary Score.

n.s.: not significant.

Z-Score: SF-36 data were normalized to a US-General population (n = 2393).

### Quantitative Sensory Testing (QST)

#### Thermal and mechanical detection thresholds

**Figure 1:**
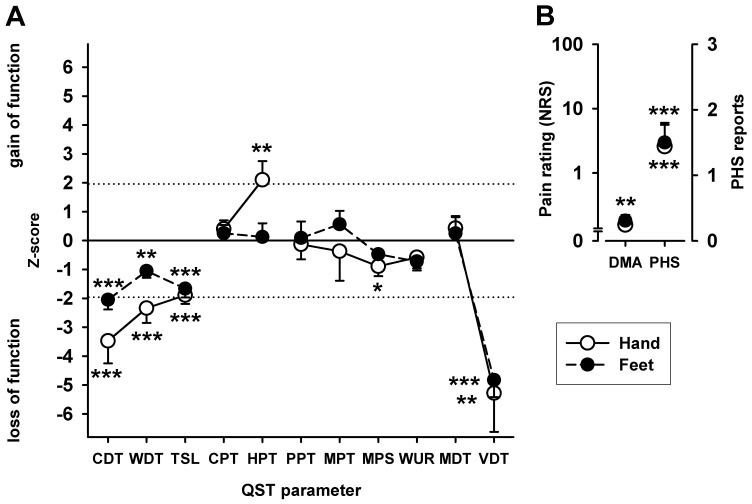
Pattern of sensory changes in patients with NMO (normalized to mean and standard deviation of healthy control group). A: The sensory profile by comprehensive quantitative sensory testing (QST) shows significant sensory loss (negative z-values) for thermal detection (CDT, WDT, TSL) and vibration detection (VDT) in both extremities, significant sensory gain (positive z-values) for noxious heat (HPT) in the hand dorsum. B: Patients with NMO experienced pronounced dysesthesia to non-noxious mechanical and thermal stimulation in both extremities, namely pain to stroking with non-noxious light tactile stimuli (dynamic mechanical allodynia DMA) and paradoxical heat sensation (PHS) to stimulation with non-noxious cold stimuli during the TSL procedure (alternating cold and warm stimuli). *p<0.05, **p<0.01, ***p<0.001, t-test.

Thermal detection (CDT, WDT, TSL) was significantly impaired in NMO patients in upper and lower extremities as compared to healthy controls (Fig. 1 and Tab.4). While healthy controls were able to identify a cooling stimulus at a temperature difference of −0.96°C in the hands (log_10_mean ± SEM: −0.017±0.032) and −1.79°C in the feet (log_10_mean ± SEM: 0.253±0.065), NMO patients detected cooling not until at a much more larger temperature change of −3.04°C (log_10_mean ± SEM: 0.483±0.107) and −7.85°C (log_10_mean ± SEM: 0.895±0.106), respectively (both p<0.001).

Similar differences were also seen for warm detection (WDT). Warming was detected at a stimulus temperature difference of +1.70°C (log_10_mean ± SEM: −0.231±0.036) in the hands and +4.31°C (log_10_mean ± SEM: 0.635±0.050) in the feet, while NMO patients detected warming only at a temperature change of +4.16°C (log_10_mean ± SEM: 0.619±0.080, p<0.001) and +7.51°C (log_10_mean ± SEM: 0.876±0.058; p<0.005), respectively. Likewise, combined alternating warm and cold stimuli (TSL) was significantly increased in the hands and feet of patients with NMO (all p<0.001, Fig. 1 and Table 4).

**Table 4:** Quantitative sensory testing (QST) in patients with neuromyelitis optica (NMO) vs. matched healthy controls (HC).

Hand		NMO^a^	HC^a^	p-value NMO vs. HC
**CDT**	Cold detection threshold (°C from BL; log) b	−3.04 (0.483±0.107)	−0.96 (−0.017±0.032)	<0.001
**WDT**	Warm detection threshold (in °C from BL; log) b	4.16 (0.619±0.080)	1.70 (0.231±0.036)	<0.001
**TSL**	Thermal sensory limen (°C; log)	9.07 (0.957±0.078)	2.74 (0.438±0.059)	<0.001
**CPT**	Cold-pain threshold (°C)	15.0±2.5	11.8±1.7	0.31
**HPT**	Heat-pain threshold (°C)	41.3±1.2	45.2±0.4	<0.01
**PPT**	Pressure-pain threshold (kPa; log)	440 (2.644±0.058)	426 (2.629±0.023)	0.82
**MPT**	Mechanical pain threshold (mN; log)	65.3 (1.815±0.134)	57.1 (1.757±0.027)	0.68
**MPS**	Mechanical pain sensitivity (pain rating 0–100; log)	0.66 (−0.181±0.106)	1.24 (0.094±0.067)	<0.05
**WUR**	Wind-up ratio (log)	1.67 (0.223±0.114)	2.49 (0.396±0.062)	0.20
**MDT**	Mechanical detection threshold (mN; log)	0.60 (−0.222±0.210)	0.99 (−0.005±0.105)	0.37
**VDT**	Vibration detection threshold (x/8)	6.10±0.44	7.82±0.07	<0.002
**DMA**	Dynamic mechanical allodynia (pain rating 0–100; log)	0.16 (−0.805±0.095)	0.00 (−1.000±0.000)	0.056
	Number of patients/subjects exhibiting DMA	5/9	0/11	<0.02^c^
**PHS**	Paradoxical heat sensations (x/3)	1.44±0.33	0.00±0.00	<0.001
	Number of patients/subjects exhibiting PHS	8/9	0/11	<0.001^c^
**Foot**		**NMO** a	**HC** a	**p-value NMO vs. HC**
**CDT**	Cold detection threshold (°C from BL; log) b	−7.85 (0.895±0.106)	−1.79 (0.253±0.065)	<0.001
**WDT**	Warm detection threshold (in °C from BL; log) b	7.51 (0.876±0.058)	4.31 (0.635±0.050)	<0.005
**TSL**	Thermal sensory limen (°C; log)	17.38 (1.240±0.071)	7.38 (0.868±0.048)	<0.001
**CPT**	Cold-pain threshold (°C)	13.2±2.5	11.0±1.9	0.49
**HPT**	Heat-pain threshold (°C)	45.6±0.8	45.9±0.4	0.76
**PPT**	Pressure-pain threshold (kPa; log)	410 (2.613±0.053)	418 (2.621±0.019)	0.89
**MPT**	Mechanical pain threshold (mN; log)	33.3 (1.523±0.127)	48.8 (1.689±0.064)	0.26
**MPS**	Mechanical pain sensitivity (pain rating 0–100; log)	0.69 (−0.159±0.074)	1.21 (0.084±0.107)	0.07
**WUR**	Wind-up ratio (log)	1.83 (0.261±0.099)	3.08 (0.489±0.066)	0.06
**MDT**	Mechanical detection threshold (mN; log)	2.92 (0.466±0.218)	3.71 (0.569±0.081)	0.66
**VDT**	Vibration detection threshold (x/8)	2.99±0.51	7.14±0.18	<0.001
**DMA**	Dynamic mechanical allodynia (pain rating 0–100; log)	0.18 (−0.741±0.090)	0.00 (−1.000±0.000)	<0.01
	Number of patients/subjects exhibiting DMA	5/11	0/11	<0.05^c^
**PHS**	Paradoxical heat sensations (x/3)	1.50±0.30	0.36±0.20	<0.005
	Number of patients/subjects exhibiting PHS	8/11	2/11	<0.05^c^

a QST-parameter; expressed as arithmetic mean ± SEM or as geometric mean (log_10_mean ± SEM; geometric mean retransformed from log_10_mean.

b Thermal detection thresholds are expressed as the difference from baseline temperature (BL = 32°C).

c Yates corrected Chi-square.

Detection of vibration (VDT) assessed by an 8/8 calibrated Rydel-Seiffer tuning fork in NMO patients was strongly reduced in the hands (6.10±0.44 vs. 7.82±0.07, p<0.002 and highly abnormal in the feet (2.99±0.51 vs. 7.14±0.18, p<0.001), where only one NMO patients exhibited vibration sensitivity in the normal range of bedside testing (>6/8) and none compared to a reference data base [18]. Mechanical detection threshold (MDT) assessed by calibrated von Frey hairs was, however, not different from healthy controls in either hands or feet (p>0.30; Fig. 1 and Tab.4).

#### Thermal Pain Thresholds

**Figure 2:**
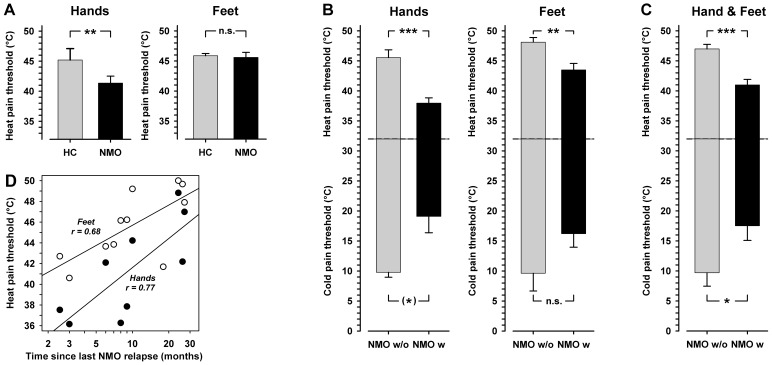
Thermal thresholds in NMO. A: Heat pain thresholds in patients with NMO (NMO) compared to healthy controls (HC) differed significantly in the hands, but not feet. **B**: Patients with NMO (NMO) in patients with an acute MRI-verified cervical lesion (NMO w) were significantly more heat pain-sensitive in both extremities and also tended to be more cold pain-sensitive than patients without (NMO w/o). For both thermal pain modalities the hyperalgesia tended to be more pronounced in the hands than feet. **C**: Collapsing data from both extremities revealed that patients with an acute MRI-verified cervical lesion (NMO w) were significantly more cold pain-sensitive than patients without (NMO w/o). **D**: Correlations of heat pain thresholds to the time span since the last relapse of an acute NMO attack was high in the hand (closed circles; r = 0.77) and feet (open circles; r = 0.68). This correlation also persisted at the same level, when normalized for gender and age. The correlations indicated that NMO relapses may have induced a severe heat hyperalgesia that subsided slowly during the course of remission. ^(^*^)^p<0.10, *p<0.05, **p<0.01, ***p<0.001, t-test.

Cold pain thresholds (CPT) did not differ significantly between NMO patients and healthy controls in hands and feet (both p>0.30). In contrast, heat pain threshold (HPT) was significantly decreased in the hand (41.3±1.2 vs. 45.2±0.4°C, p<0.01), indicating significant heat hyperalgesia. Heat pain thresholds in the feet, however, were unaltered (45.6±0.8 vs. 45.9±0.4°C, p = 0.76 Fig. 2A and Tab.4).

In order to elucidate these obvious differences in heat pain thresholds between the upper and lower extremities, we stratified NMO patients according to the presence of MRI-verified structural cervical spinal lesions (six patients; # 4, 5, 6, 7, 8, 11) excluding those without cervical lesions (five patients; # 1, 2, 3, 9, 10). While patients without cervical spinal lesion did not differ from healthy controls at all regarding their heat pain threshold (45.5±1.3 vs. 45.2±0.4°C, p = 0.84), patients with structural cervical lesions displayed a highly significantly reduction of heat pain threshold (heat pain hyperalgesia), as compared to control group at the upper extremities (38.0±0.9°C, p<0.001 vs. healthy controls and vs. NMO patients without cervical lesions; Fig. 2B). A high correlation between thermal pain thresholds assessed in the hand and foot dorsum (r = 0.81 and r = 0.71 for cold and heat pain, respectively) prompted a similar comparison for the feet. Although there was no overall difference of heat pain threshold between NMO patients and healthy controls in the feet (p = 0.78), patients with a cervical lesion had nevertheless significantly lower heat pain thresholds than those patients without (43.5°C ±1.1 vs. 48.1±0.8°C, p<0.005) suggesting that cervical lesions may also affect spinothalamic tract fibers of passage from the lower extremities running in the anterolateral tract (Fig. 2B).

Similar trends were observed for cold pain thresholds (Fig. 2B), and pooled data from the hands and feet revealed that cold pain thresholds in NMO patients without signs of cervical lesion (9.7±2.2°C) were similar to control subjects (11.40±1.3°C, p = 0.51), while in patients with signs of a cervical lesion cold pain thresholds were significantly shifted to higher temperatures (17.5±2.4°C, p<0.05 vs. patients without lesions and vs. healthy controls; Fig. 2C). In contrast, there was a complete lack of differences in mechanical pain thresholds (p = 0.48 for PPT, and p = 0.73 for MPT).

Comparing both subgroups regarding their NMO history revealed that patients with MRI-identified acute inflammatory lesions were significantly closer to their last relapse of NMO (6.2 vs. 16.4 months, p<0.05). Consequently, there was a highly significant correlation of heat pain thresholds with time since last relapse of NMO for both hand and feet (r = 0.77 and r = 0.68, both p<0.01), which reached normal thresholds not until one year after the last NMO relapse (Fig. 2D).

#### Paradoxical heat sensations

PHS was a very frequent finding in TSL trials with NMO patients at either the examined hands or feet. Overall, PHS was detected in 8/11 patients with NMO, and 33 of 66 trials (50%) at the feet, but only in 8 of 66 trials at the feet of healthy controls (12.1%, p<0.01). Moreover, whereas none of the healthy controls showed PHS in any trial at the dorsum of their hand, PHS regularly occurred in 8/9 patients with NMO at the hands, and was experienced in 26 of 66 trials, (39.4%, p<0.001; Tab.4 and 5).

### Mechanical Pain Thresholds

Concerning the mechanical pain thresholds, QST testing revealed no signs of mechanical hyperalgesia. Neither mechanical pain thresholds (MPT), pressure pain threshold (PPT) nor wind-up ratio (WUR) following pin-prick stimulation differed significantly in any extremity of NMO patients compared to healthy controls (p>0.05, each, Tab.4). In addition, pain sensitivity for pinprick stimuli (MPS) was significantly reduced in the hand of NMO patients (p<0.05), accompanied by a strong trend towards MPS reduction in the feet (p = 0.07; Table 4). This trend was approved when comparing QST testing for MPS in all extremities with healthy controls (p<0.001). However, pain to stroking light touch (Dynamic Mechanical Allodynia DMA) was present in NMO patients in both extremities compared to healthy controls as well as to DFNS reference data (Hands: p = 0.056; Feet: p<0.01 Tab.4; both extremities: p<0.001).

### Frequency of Somatosensory Loss or Gain

**Figure 3:**
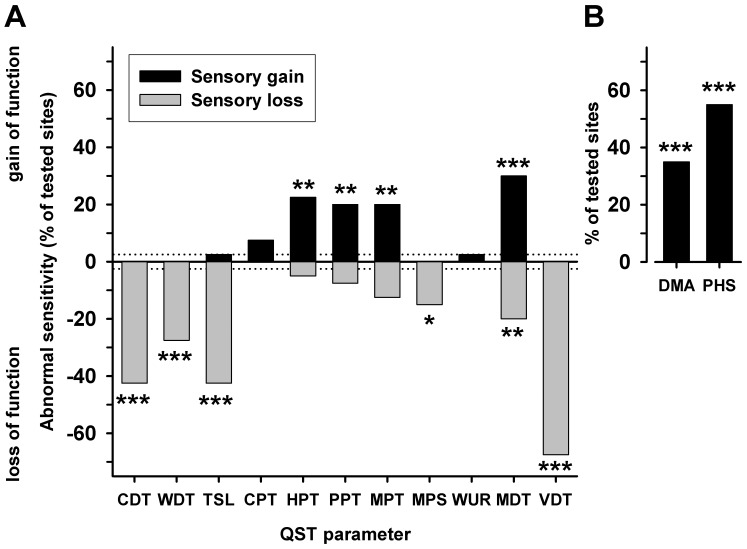
Abnormal somatosensory findings in NMO. **P**attern of abnormal somatosensory findings in patients with NMO encompassing sensory gains (black bars) and sensory losses (grey bars). Highly significant loss of sensitivity was prevalent for all non-nociceptive detection (except for tactile detection MDT, which exhibited also a similar number of abnormal gains in sensitivity). Highly significant gain of sensitivity was prevalent for all pain thresholds (except for cold pain threshold CPT) and for the painful dysesthesias elicited by non-noxious stroking tactile stimuli (dynamic mechanical allodynia DMA) and heat pain sensation elicited by non-noxious cold stimuli during the TSL procedure (paradoxical heat sensation PHS). Healthy controls did not exhibit abnormal frequencies in any of the 13 QST parameters. *p<0.05, **p<0.01, ***p<0.001, Yates-corrected Chi^2^-test.

Somatosensory parameters were judged based on an age and gender adjusted reference data base of healthy subjects [18]. Healthy control subjects displayed a statistically normal distribution, with 7/242 tests for the hands (2.9%) and 9/242 tests for the feet (3.7%) outside the 95% confidence interval not exceeding the expected 5% rate. In contrast, abnormal sensitivity was frequent in NMO patients and occurred in 11/13 somatosensory parameters (all except CPT, WUR; Fig. 3) with similar rates for hand or feet (81/198 = 40.9% and 105/242 = 43.4%, respectively) and similar proportions of somatosensory loss or gain (99/440 = 22.5% and 87/440 = 19.8%, respectively). Overall, NMO patients exhibited significantly more abnormal sensory loss than gain in non-nociceptive QST parameters (loss: 81/200 = 40.5%>>gain: 14/200 = 7.0%; p<0.001), but significantly more abnormal gain of nociceptive sensitivity and dysesthesia than loss (gain: 65/240 = 27.1% >>. 18/240 = 7.5%; p<0.001; Fig. 3).

In aggregate, the comprehensive QST revealed three main features: First, patients with NMO displayed a highly significant thermal sensory loss indicated by the increase of thermal detection thresholds (CDT, WDT and TSL). Mechanical detection thresholds were abnormal in 50% of patient test sites, but remained relatively unchanged overall, since abnormal sensitivity encompassed a similar proportion of abnormal gains or losses (Fig. 3). Additionally, we found a pronounced loss of vibration sensitivity.

Secondly, patients with NMO exhibited thermal hyperalgesia (to painful heat and cold stimuli), when they exhibited signs of cervical spinal lesion as a marker of inflammatory spinal cord damage. The amount of hyperalgesia was strongly correlated to the time since the last relapse of NMO. In contrast, mechanical pain parameters did not reveal clear signs of hyperalgesia.

Thirdly, NMO patients displayed distinct signs of dysesthesia in both hands and feet, namely the frequent occurrence of paradoxical heat sensations following alternating warm/cold stimulation, and significant pain to light touch.

Finally, the magnitude of on going pain at the time of QST assessment was strongly negatively correlated to any of the QST pain parameters with no difference between thermal and mechanical pain modalities (average r = 0.73, p<0.01; range: r = −0.51 to −0.80) suggesting a deafferentation-type of central neuropathic pain.

### Plasma Levels of Endogenous Cannabinoids

**Figure 4:**
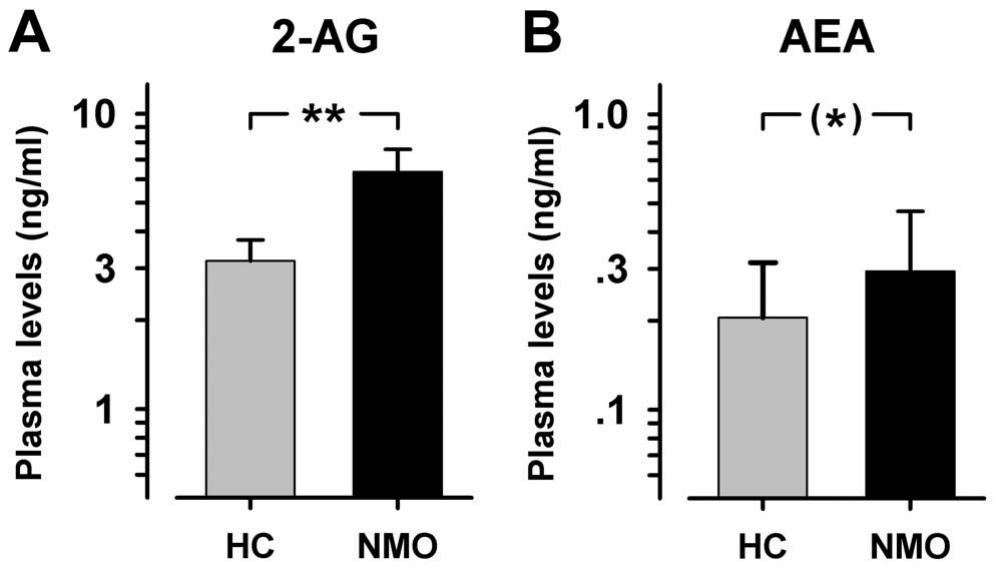
Endocannabinoid plasma levels in NMO. Mean plasma levels of the endogenous cannabinoid lipids 2-arachidonoylglycerol (2-AG) and N-arachidonoylethanolamide (AEA, anandamide) were increased in patients with NMO compared to age matched healthy controls. (*)p<0.10, **p<0.01.

The two major chemically related cannabinoid lipids anandamide and 2-arachidonoylglycerol (2-AG) were elevated in patients’ plasma compared to age and gender-matched healthy controls. 2-AG plasma levels were doubled (mean 6.37 vs. 3.18 ng/ml; log10∶0.804±0.075 vs. 0.502±0.071, p<0.01), while anandamide exhibited only a trend (increased by 44%; mean 0.295 vs. 0.205 ng/ml; log10∶0.531±0.061 vs. 0.688±0.056, p = 0.07) (Fig. 4). While plasma levels of anandamide and 2-AG were highly correlated in healthy controls (r = 0.69, p<0.01), they were fully uncoupled in patients with NMO (r = 0.09, n.s.).

**Figure 5:**
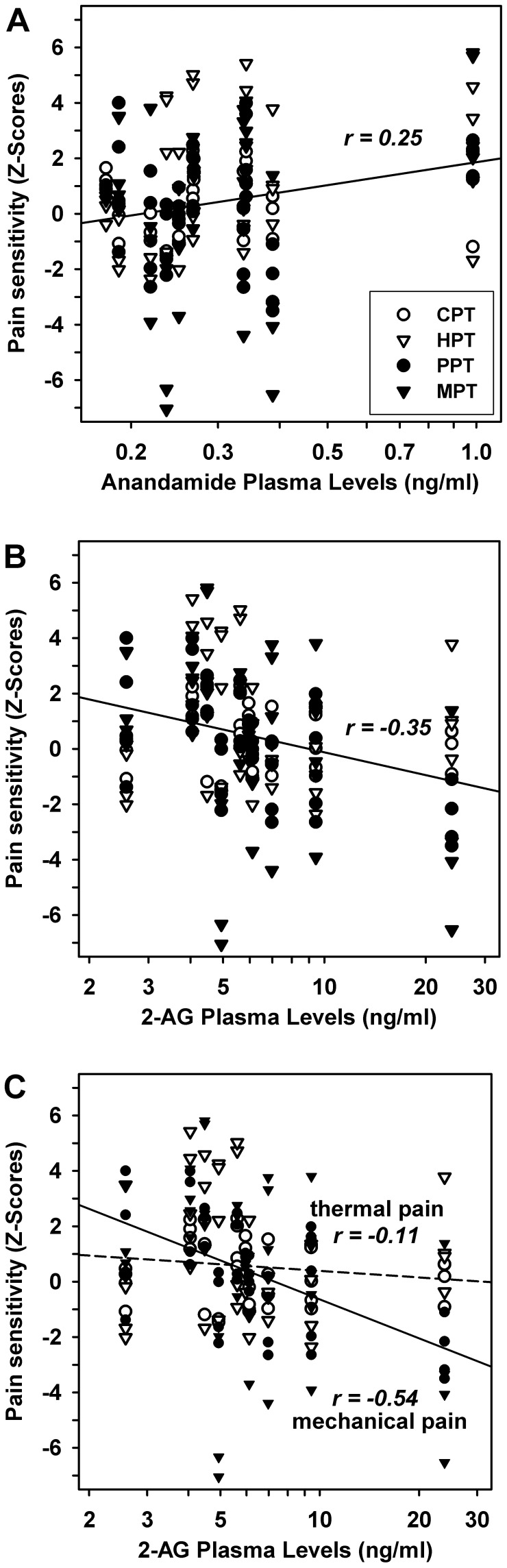
Correlations between pain sensitivity and endocannabinoid plasma levels. The figure displays correlations of quantitative sensory testing of pain sensitivity (normalized pain thresholds) to plasma concentrations of the cannabinoid lipids *N*-arachidonoylethanolamide (AEA, anandamide) and 2-arachidonoylglycerol (2-AG) in patients with NMO. Therefore, the results of all QST parameters testing mechanical (PPT, MPT) or thermal (CPT, HPT) pain sensitivity in each patient and each test site (both hands and feet) were analysed. **A**: The plasma concentration of AEA was weakly positively correlated to normalized pain thresholds collapsing estimates in the hand and feet dorsums. **B**: The plasma concentration of 2-AG was significantly negatively correlated to normalized pain thresholds. **C**: Correlation of 2-AG concentrations to normalized pain thresholds in hand and feet dorsums differed significantly between thermal (no correlation) and mechanical pain thresholds (substantial negative correlation). The latter varied by more than four standard deviations between the lowest and highest concentration of plasma 2-AG in an NMO patient. Thermal pain thresholds (open symbols), mechanical pain thresholds (closed symbols).

Both, 2-AG and anandamide were not significantly related to the time since last relapse (r = −0.07 and r = −0.22, both p>0.50). In contrast, there were significant relationships of both lipid signalling molecules to the patients’ somatosensory status. Anandamide levels were positively, but weakly correlated to pain sensitivity as calculated by normalized pain thresholds (r = +0.25, p<0.02; Fig. 5A) with no difference between thermal (CPT, HPT) and mechanical pain thresholds (PPT, MPT). In contrast, 2-AG levels were negatively correlated to pain sensitivity (r = −0.35, p<0.001; Fig. 5B). Thus, anandamide and 2-AG were significantly different in their correlation pattern to pain sensitivity (p<0.001; Fisher’s z), also when calculated separately for upper and lower extremities (p<0.001 and p<0.01; Fisher’s z). At closer inspection, the correlation between 2-AG and pain thresholds was present for mechanical pain thresholds (r = −0.54, p<0.001; r = −0.49 for MPT and r = −0.61 for PPT), but not thermal pain thresholds (r = −0.09 for CPT and r = −0.13 for HPT, both n.s.; Fig. 5C). In contrast, correlations to non-nociceptive detection thresholds were variable and overall no significant relationship to cannabinoid levels was found (r = −0.12 and r = +0.09, respectively, n.s.).

## Discussion

Central neuropathic pain (CNP) is a frequent, but often neglected phenomenon in various forms of CNS diseases such as NMO. Notwithstanding, mechanisms underlying this type of pain are poorly understood. Accordingly, pain has recently been described as another key symptom of NMO, but the predominant quality of pain has not yet been characterized [10], [11].

### Pain Characteristics, Stress, Depression and Health Related Quality of Life in NMO

As almost all patients reported pain at the time or in the three months preceding the examination, our data emphasize the high incidence of pain also in Caucasian NMO patients compared to patients with multiple sclerosis (28%−47%) or spinal cord injury (30−40%)[11], .

The pain characteristics in NMO patients were burning or tingling, sensory descriptions discriminating neuropathic from non-neuropathic pain states [31]. Notably, on going pain at the time of QST assessment correlated negatively with any of the QST pain parameters regardless of the respective pain modality, thereby suggesting a deafferentation-type of central neuropathic pain [47], [48].

Similar to previous findings, we consistently found significantly reduced scores in three of the eight dimensions of the SF 36 reflecting impaired Health Related Quality of Life, [10], [49], and distinct signs of depression even exceeding those found in patients with chronic Complex Regional Pain Syndrome (CRPS), where depression was substantially correlated to the level of ongoing pain [50]. However, levels of stress were not considerably increased in NMO patients.

Consequently, we strongly recommend the routine use of supplementary questionnaires amending the EDSS, in order to achieve timely recognition and treatment of pain as well as depression in NMO.

As the pattern of pain suggested a neuropathic origin of the ongoing pain in patients with NMO, in the next step we used QST in order to delineate the accompanying somatosensory changes and to get insights into the pathophysiological background of neuropathic pain in NMO as recently published for a n>1200 cohort of neuropathic pain patients [15].

### Somatosensory Changes in Neuromyelitis Optica - Negative Sensory Signs

In NMO patients, the ability to detect either warm or cold was considerably impaired, indicated by the threefold increase in CDT, WDT and TSL. The occurrence of negative thermal sensory signs is characteristic for patients with all kinds of neuropathic pain disorders, regardless of a peripheral or central site of lesion [15]. As NMO typically generates extensive spinal cord lesions, thermal sensory loss is most likely related to lesions of cell bodies in the spinal dorsal horn or their axons projecting into the ascending spinothalamic tract [51], [52], which represent a major cause of central neuropathic pain after spinal cord injury [53]. Increased thermal sensory thresholds are associated with the development of central neuropathic pain [54], [55], especially when some residual spinothalamic tract function was preserved to constitute a central pain generator [56]. Likewise, in partial nerve lesions degenerating nerve fibers cause hyperexcitability and ectopic discharge of remaining intact axons [57]. As spinal cord injury is mostly incomplete in the majority of NMO patients [58], a fractional impairment of the spinothalamic tract may constitute one important generator of neuropathic pain in NMO.

Similarly, a pronounced impairment of vibration detection was observed, which is a characteristic of somatosensory changes in patients with neuropathic pain as well [15], [50], [59]. Admittedly, mechanical detection thresholds appeared to be unaffected, as no significant increase of MDT compared to a healthy control or the DFNS cohort of healthy subjects could be detected (Table 4, Fig. 1). However, the pattern of abnormal somatosensory findings (Figure 3) revealed a significant loss of tactile detection in more than 25% of the patients, which was counterbalanced by a gain of mechanical detection in approximately the same proportion of different patients. However, a disturbed function of the dorsal column medial lemniscal pathway seems not to be necessary for the development of central pain in stroke patients [60].

### Somatosensory Changes in Neuromyelitis Optica - Positive Sensory Signs

There was apparently no overall mechanical hyperalgesia to pinprick or blunt pressure. However, approximately 20% of the patients nevertheless displayed abnormal mechanical hyperalgesia, yet balanced by patients with mechanical hypoalgesia (Fig. 3). Importantly, on closer examination we found a considerable negative correlation between the plasma levels of 2-AG and the mechanical pain thresholds (discussed below).

The most conspicuous positive mechanical sign was dynamic mechanical allodynia (DMA), i.e. pain to stroking light touch stimuli, which marks a crosstalk of tactile inputs into pain pathways, and is considered as a hallmark sign of central sensitization [61]–[63]. Beside spinal lesions, neuronal hyperexcitability has been proposed to be a necessary requirement for the development of neuropathic pain in patients with spinal cord injury [64]. Interestingly, DMA was nearly twice more frequent (35%) than observed in the DFNS cohort of neuropathic pain patients (19.7%), and was particularly more frequent than in the subgroup of patients suffering from other forms of central neuropathic pain (17.7%) [15].

However, the low incidence of abnormal pain sensation (wind up pain sensation), despite clear signs of central sensitization is not contradicting the finding of central nociceptive sensitization, as central sensitization and wind-up represent independent phenomena [65], [66]. Moreover, this is in line with the low incidence of pathological wind-up in the DFNS cohort (12%) in contrast to the considerably higher incidence of mechanical hyperalgesia (20–40%) [15].

Hyperalgesia to noxious heat or cold is usually attributed to peripheral sensitization of primary afferent C fibers, a hallmark sign of inflammatory processes [67], [68]. Interestingly, thermal hyperalgesia, especially to noxious heat, depended on the presence of a spinal lesion and was particularly severe immediately after a NMO relapse, which is known to cause inflammatory demyelization of the spinal cord [69], [70]. Central neuroinflammation is supposed to be one key mediator of thermal hyperalgesia and allodynia in experimental autoimmune encephalitis [71]. Additionally, after experimental spinal cord injury, the development of central neuropathic pain was associated with the upregulation of several proinflammatory cytokines [72] as well as other proteins such as S100ß, glial fibrilary acidic protein (GFAP), and AQP-4 [73], the latter representing the target antigen in NMO [7]. Elevation of these proteins persisted for at least 9 months in rodents with central neuropathic pain, while animals that did not develop central neuropathic pain after spinal cord injury did not up-regulate AQP-4 neither in the acute nor in the chronic stage of the disease [73]. Therefore, we propose that the degree of thermal hyperalgesia corresponds to the current level of NMO-associated neuroinflammation, which can be identified using QST. Finally, paradoxical heat sensations (PHS), i.e. mild cold stimulus erroneously perceived as hot or burning pain, represents a disturbance of integration in thermosensory pathways, and is hardly ever present in healthy subjects. The incidence of PHS in this study (8/11 patients = 73%) was considerably higher than in patients suffering from any other form of central pain (26%) as well as in the entire DFNS cohort of patients with neuropathic pain (18.4%) [15]. Importantly, PHS is also a frequent finding in multiple sclerosis [74]. Mechanistically, the occurrence of PHS is promoted by central disinhibition [75], facilitated by combined inflammatory and neurodegenerative processes [76], the latter likely also explaining the high incidence in NMO.

### Proposed Role of Endocannabinoids in the Pathophysiology of Pain in NMO

In animal models of neuropathic pain, gliopathy, involving microglia in the early stages and astrocytes in the later stages, parallels persistent pain in spinal cord injury [77]–[79]. After a few weeks, astrocytes critically maintain persistent pain, and astrocytic networks are capable of transducing signals for extended distances across and along the spinal cord [80]. We propose that spinal astrocytes may also be key regulators of pain sensitivity in NMO patients. Glia is an important cellular component of synaptic plasticity involving the release of endogenous cannabinoids, and we hypothesized that differing cannabinoid release from astrocytes may explain the wide variability of individual responses encountered in central types of neuropathic pain. Detailed single patient analysis revealed abnormal hyperalgesia, as well as hypoalgesia with a wide variation of almost five standard deviations of the healthy reference cohort. Post-hoc correlation analysis revealed that this wide interindividual variation of mechanical pain sensitivity in individual patient pain phenotypes could be partially explained by the level of the major endogenous brain cannabinoid lipid 2-AG. 2-AG is known to inhibit synaptic transmission by at least three major mechanisms, namely retrograde negative feedback at glutamatergic synapses acting presynaptically as a synaptic circuit breaker by downregulating transmitter release [28]. Furthermore, at physiologically concentrations, 2-AG strongly enhances GABAergic inhibition by allosteric modulation of the GABA-A receptor [29]. Furthermore, 2-AG is the major brain cannabinoid acting at CB1 cannabinoid receptors, which are important regulators of homeostatic neuronal plasticity [30]. Very recently it has been shown that activation of the CB1 cannabinoid receptor, which is the target of 2-AG released by astrocytes, promotes the expression of synaptic long-term depression in the spinal cord and neocortex involving the activation of nearby astrocytes [81], [82]. The autoimmune attack on AQP-4 abundantly located on astrocytes induces excitation and subsequent degeneration through complement mediated cytotoxicity causing a lack of inhibitory tone on adjacent neurons [83]. Demyelination by oligodendrocyte apoptosis subsequent to loss of trophic support from astrocytes leads to extensive axonal injury causing severe disability [84]. Additionally, degeneration of astrocytes impairs astrocyte-mediated control of the blood-brain-barrier [85], [86], thus allowing for the detection in systemic circulation of 2-AG, which otherwise does not cross the blood-brain-barrier and may under these conditions mirror cerebral concentrations. Consequently, increasing plasma levels of 2-AG (up to ten fold of normal concentrations) mitigate mechanical pain sensitivity, while an absence of 2-AG increase leaves these NMO patients with full-blown hyperalgesia. Similar findings were made for other aversive syndromes [87], [88] Notably, the intrinsic up-regulation of 2-AG in neurons and astrocytes represents a protective system preventing secondary neuronal damage and promoting neuronal survival [89].

### Study Limitations

Currently, there is no comprehensive characterization of the interaction between analgesic medication and somatosensory testing and we cannot rule out interference with analgesic treatment. However, no influence of antidepressant medication on thermal thresholds could be found in patients suffering from major depression [90]. The relative small sample size due to the rarity of the disease and anatomical heterogeneity of the lesions warrant cautious interpretation of results. However, we adjusted for this bias by using standardized test sites as suggested by the DFNS, viz. the dorsum of the hand and feet, and comparing with a healthy control group adjusted for confounding factors like age and gender [18]. Nevertheless, a multicenter study and larger number of patients or a tailored control group for instance with patients suffering from defined anatomical lesions of the spinal cord, are mandatory to confirm the results.

In aggregate, patients with NMO are displaying both the clinical as well as the typical somatosensory signs of neuropathic pain. The “unhappy” trias of central lesions, accompanied by central sensitization of pain pathways and contemporaneous CNS inflammation in NMO accounts for the high incidence of neuropathic pain. Additionally, the pain phenotype appears to be modulated by 2-AG, the major endogenous cannabinoid of the brain**,** for which we propose a double inhibitory role on both mechanical hyperalgesia and potentially also on neuroinflammation.
